# Efficacy of Extracorporeal Shockwave Therapy in the Management of Chronic Diabetic Foot Ulcer: A Systematic Review and Meta-Analysis

**DOI:** 10.3390/medsci13040219

**Published:** 2025-10-02

**Authors:** Maria Ruiz-Muñoz, Lidia Rueda-Zapata, Francisco-Javier Martinez-Barrios, Tereza Nováková, Eva Lopezosa-Reca, Manuel Gonzalez-Sanchez, Raul Fernandez-Torres, Alejandro Galan-Mercant

**Affiliations:** 1 Department Nursing and Podiatry, Faculty of Health Sciences, University of Málaga, 29004 Málaga, Spain; marumu@uma.es (M.R.-M.); lidia_rz@uma.es (L.R.-Z.); evalopezosa@uma.es (E.L.-R.); fernandeztorres@uma.es (R.F.-T.); 2 Department of Physiotherapy, Faculty of Physical Education and Sport, Charles University, 16252 Prague, Czech Republic; tnovakova@ftvs.cuni.cz; 3 Department of Physiotherapy, Faculty of Health Sciences, University of Málaga, 29004 Málaga, Spain; mgsa23@uma.es; 4 Department Nursing and Physiotherapy, Faculty of Health Sciences, University of Cadiz, 11009 Cádiz, Spain; alejandro.galan@uca.es

**Keywords:** extracorporeal shockwave therapies, diabetic foot disease, meta-analysis, ulcer treatment, DFU, diabetic wound, diabetic foot ulcer

## Abstract

**Introduction:** This study will explore the effectiveness of current extracorporeal shockwave therapies in ulcer healing in diabetic foot disease compared to the standard of care. **Methods:** The systematic review and meta-analysis were performed following the Preferred Reporting Items for Systematic Reviews and Meta-Analyses (PRISMA) standard. The electronic databases WoS, EMBASE, MEDLINE Complete, CINAHL Complete, Academic Search Ultimate, AMED-The Allied and Complementary Medicine Database, Scopus and PubMed searched for the outcome rate of complete ulcer healing. The risk of bias assessment was conducted using the tool recommended by the Cochrane Collaboration (Robvis Tool). Statistical analysis included the individual and combined result of the studies, heterogeneity test, the effect size, sensitivity analysis, and publication bias tests. **Results:** Eight randomized controlled trials (RCTs) with a total of 672 patients were included in this study. This meta-analysis showed a higher rate of complete ulcer healing in groups receiving extracorporeal shockwave therapies (OR = 2.747 [1.965, 3.841], *p* < 0.01, I^2^ =0.02) compared to control groups. **Conclusions:** In conclusion, the results of this meta-analysis show that extracorporeal shockwave therapies, when used as an adjunctive treatment, demonstrate significantly higher ulcer healing rates compared to standard of care. Extracorporeal shockwave therapies should be taken into account as a valuable adjunct to standard care for diabetic foot ulcer treatment.

## 1. Introduction

Diabetes is a chronic metabolic disorder characterized by elevated blood glucose levels resulting from the body’s inability to produce or effectively use insulin. It is a growing global public health problem and affects approximately 463 million adults worldwide [1].

Among the most common complications of diabetes are diabetic foot ulcers (DFUs). DFUs are defined as lesions that involve a break in the skin with loss of epithelium: they can extend into the dermis and deeper layers, sometimes involving bone and muscle. It has been estimated that the lifetime risk of a person with diabetes developing a foot ulcer is as high as 30–34% [2,3]. Up to 40% of DFU patients will suffer major or minor amputations, and 70% of these people will die within 5 years of amputation [4,5]. In 2008, the US spent $18 billion on DFU care and $11.7 billion on lower limb amputations [6].

Furthermore, the type of ulcer can be classified according to its ethology as neuropathic, neuroischemic, ischemic or pressure ulcer. Depending on this classification, the treatment will be oriented. The management of DFUs ranges from infection control to restoration of tissue perfusion [7,8].

Extracorporeal shock wave therapy (ESWT) is a non-invasive intervention that is gaining ground in the treatment of DFUs. Given the high prevalence of diabetes and the high rate of DFU, it is crucial to address this problem and to know the most effective treatments. Early detection and treatment of complications can improve outcomes and reduce the risk of serious complications.

It aims to stimulate healing by creating shear forces in the tissues. ESWT is indicated for lesions with delayed healing, burns, venous and arterial ulcers, ultra-pressure ulcers and diabetic patients [9,10].

There is evidence that ESWT promotes wound healing in soft tissues. There are multiple hypotheses about the mechanism of its therapeutic effect, the first hypothesis indicates that ESWT may act by directly disrupting and remodeling plaques; the second hypothesis proposes that ESWT may generate heat locally, leading to an active inflammatory response with increased macrophage activity and subsequent plaque dissolution and absorption [10].

The most recent meta-analysis on shock wave therapies analyzing the healing rate of chronic ulcers in diabetic foot patients includes randomized clinical trials up to the year 2014. The current literature features new clinical trials that render the latest meta-analysis outdated, making it necessary to analyze the new body of evidence in an updated meta-analysis. Thus, this study will be conducted with the objective of evaluating the effectiveness of ESWT as a complementary therapy to standard of care (SOC) for the treatment of chronic DFU. With the current available evidence from the literature, will analyze whether the healing rate with ESWT is higher than with SOC.

Keywords: Extracorporeal shockwave therapy, diabetic foot disease, meta-analysis, ulcer treatment, randomized controlled trials.

## 2. Material and Methods

The systematic review and meta-analysis were performed following the Preferred Reporting Items for Systematic Reviews and Meta-Analyses (PRISMA) standard [11]. The protocol was prospectively registered in the International Prospective Register of Systematic Reviews (PROSPERO database CRD420250527214).

### 2.1. Eligibility Criteria

This meta-analysis investigated randomized controlled trials (RCTs) that evaluated the efficacy of extracorporeal shockwave therapy (ESWT) versus standard treatments (debridement, blood-glucose control agents, and footwear modifications) for managing diabetic foot ulcers (DFUs) that are unresponsive to conventional therapies. The review exclusively considered studies with human participants. The results were reported following the 2020 Preferred Reporting Items for Systematic Reviews and Meta-Analyses (PRISMA) guidelines [11]. To be included in the analysis, studies needed to meet specific criteria: they had to be RCTs, involve participants diagnosed with DFUs, utilize both ESWT and standard treatments, and measure the healing rate of DFUs as the primary outcome. Studies were excluded if they did not compare treatment effectiveness or if they lacked quantitative data suitable for statistical evaluation. The Population, Intervention, Control, and Outcome (PICO) framework applied was as follows: Population: Adult patients with chronic DFUs; Intervention: ESWT; Control: Standard of care (SOC); Outcome: Healing rate.

To identify the evidence, the WoS, EMBASE, MEDLINE Complete, CINAHL Complete, Academic Search Ultimate, AMED—The Allied and Complementary Medicine Database, Scopus and PubMed databases were searched. The last search was performed in July 2024. The search was performed using the keywords “Diabetic foot ulcer”, “DFU”, “ extra corporeal shockwave therapy”, “extracorporeal shockwave therapy”, “ESWT”, “diabetic wound”, using the Boolean operators “AND” and “OR”: ((Diabetic foot ulcer) OR (DFU) OR (diabetic wound)) AND ((extra corporeal shockwave therapy) OR (extracorporeal shockwave therapy) OR (ESWT)).

### 2.2. Selection Process, Data Collection, and Data List

Two reviewers (A.M.R. and F.M.B) independently performed a double-blind evaluation of titles and abstracts to determine their eligibility based on the inclusion criteria, achieving a Cohen’s kappa of 0.83. In cases of uncertainty, the full texts were reviewed, yielding a Cohen’s kappa of 0.88. Any disagreements were discussed to reach a resolution; if consensus could not be achieved, a third reviewer (M.R.M) was involved. Although it was planned to contact the original authors for further details if needed, this step was not required.

The primary reviewers independently created two tables to extract data from the eligible studies. Extracted information included the author’s name, publication year, country, participant numbers, gender, age, hemoglobin glicosilade (HbA1c), body mass index (BMI), ulcer size, diabetes diagnostic, ulcer classification, treatment strategy, application ESWT, time treatment, healing rates, and conclusions. Data regarding any outcome related to DFU healing rates in both the experimental and control groups were collected.

Each article underwent a separate risk of bias assessment conducted by the main reviewers using the Robvis tool, as recommended by Cochrane [12]. Additionally, the studies were methodologically evaluated using the Spanish Critical Appraisal Skills Program (CASPe). Studies scoring below eight out of eleven points were excluded from the analysis [13].

### 2.3. Statistical Analysis

The evaluation employed the log odds ratio to measure outcomes, with results later converted back to odds ratio. A random-effects model was utilized for the dataset. Statistical analysis followed a dichotomous approach, using a random-effects model to compute the log odds ratio (LogOR) and then back-transforming it to odds ratio (OR) with a 95% confidence interval (CI = 95%) [14]. Heterogeneity among studies was assessed using measures such as I2, the Q statistic, and H2 [15]. The I2 statistic ranges from 0% to 100%, where 0% indicates no heterogeneity, less than 25% indicates low heterogeneity, 25–50% indicates moderate heterogeneity, 50–75% indicates high heterogeneity, and 75% or more indicates very high heterogeneity. A significance level of 10% rather than 5% is recommended for the Q statistic. Complete uniformity is indicated by H = 1, with a theoretical range from one to infinity. Heterogeneity is considered significant at the 5% level if the 95% confidence interval for the H statistic does not include the null value (one). There is no standard for classifying H2 as mild, moderate, or severe [16].

Rosenthal’s method [17] was utilized to estimate the number of additional studies needed, with an average null result, to reduce the combined significance to a specified α level (typically 0.05). To complete the bias assessment, the rank correlation test was performed to determine if there was a relationship between the observed effect sizes or outcomes and their respective sampling variances. A strong correlation would suggest asymmetry in the funnel plot, which could indicate publication bias [18].

Additionally, a Galbraith plot was constructed to assess the level of heterogeneity among the studies in the meta-analysis. In this plot, the y-axis shows the (log-transformed) effect size divided by its standard error (z score), while the x-axis represents the inverse of the standard error [19]. A L’Abbé plot was also used to examine heterogeneity among studies, plotting the risks (or odds) of the exposed or index group on the y-axis against those of the control group [20]. Publication bias was initially assessed through sensitivity analysis and an influence graph. This involved replicating the meta-analysis results while omitting one study at a time, aiming to verify the consistency of outcomes in terms of direction, effect size, and statistical significance, thereby demonstrating the robustness of the analysis [21]. If statistically significant bias was found (*p* > 0.1), a “trim and fill” analysis was performed to estimate the number of additional studies needed to correct for publication bias, and the revised effect size was compared with the original to check for significant differences [22].

Data analysis and statistical calculations were conducted using SPSS v.28, EpiDat v.3.1, RevMan v.5.4.1, and Jamovi v.2.3.28.0 software packages.

## 3. Results

### 3.1. Study Selection

**Figure 1:**
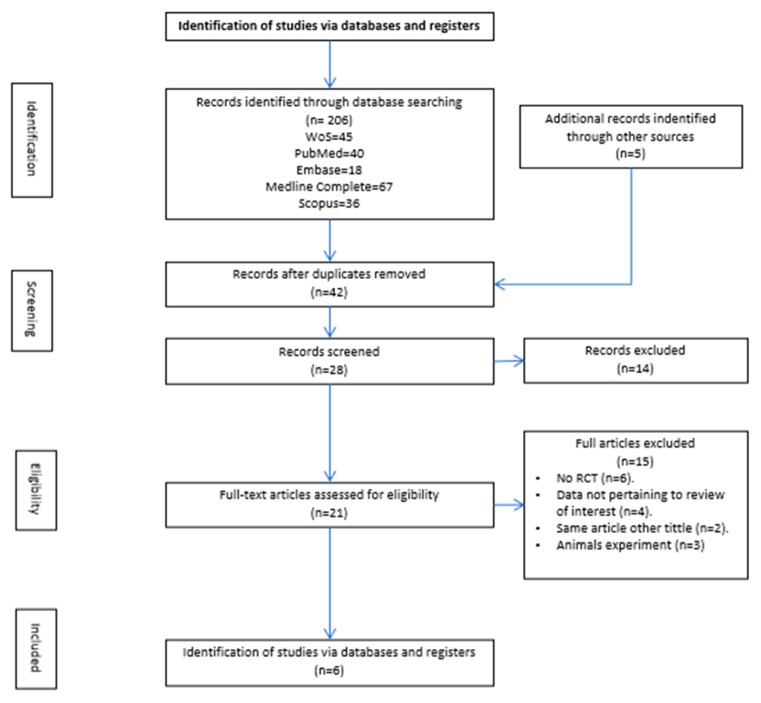
PRISMA (2020) flowchart detailing information flow throughout the review process.

The study selection process was illustrated using the PRISMA flow diagram (Figure 1). Initially, 206 articles were identified, which were then reduced to 21 articles for full-text review and eligibility assessment. Finally, 8 articles were included in this review (Figure 1). These selected studies underwent a risk of bias evaluation, with satisfactory outcomes. Additionally, a critical appraisal using the CASPe tool indicated high methodological quality.

The included studies comprised a total of 336 individuals in the experimental group and 254 individuals in the control group, amounting to a total of 672 participants (Table 1).

The quality of the studies was evaluated using the CASPe tool, which is specifically designed for critical assessment of scientific evidence [23,24,25,26,27]. All eight clinical trials included in this analysis were reviewed, each receiving scores 10 and 11 (Table 2), indicating a high standard of quality.

**Figure 2:**
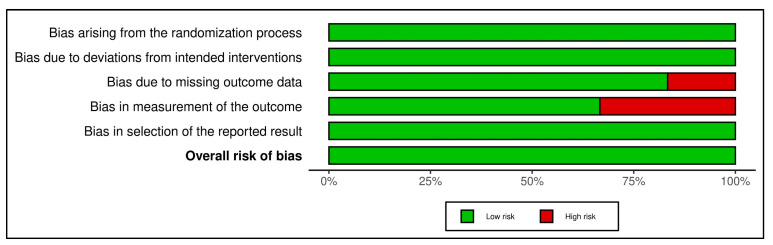
Risk of bias graph: review authors’ judgements about each risk of bias item presented as percentages across all included studies.

**Figure 3:**
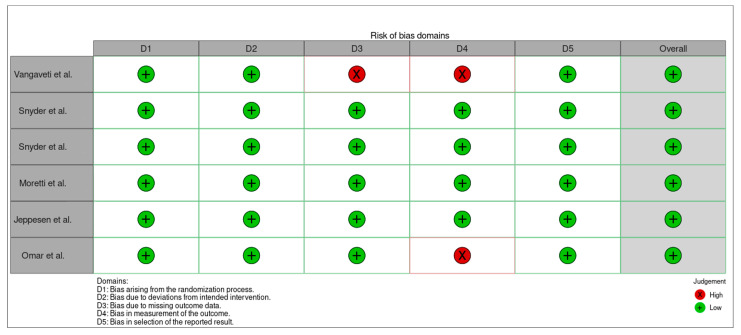
Risk of bias summary: review authors’ judgements about each risk of bias item for each included study [23,24,25,26,27].

The risk of bias assessment followed the Cochrane Collaboration guidelines (Robvis) [12], using a tool that classifies bias into five categories, each with specific domains: selection bias involves four domains, performance bias and detection bias each cover one domain, attrition bias includes two domains, and reporting bias is addressed within a single domain (Figure 2 and Figure 3).

### 3.2. Results of Individual Studies

The final analysis of our meta-analysis included a total of 6 studies that met the inclusion criteria. These clinical trials provided data for a total of 672 patients, lending considerable strength to our findings. When evaluating the contribution of each study to this result, we found that the trials by Snyder [24] had the largest weight, underscoring the importance of their findings given their large sample size. In contrast, the study by Jeppesen [26] had the least contribution, indicating its minor influence on the results due to its smaller sample size (Table 3).

### 3.3. Effect Size Estimation and Primary Outcomes

Table 3 presents the effect sizes (Log Odds Ratio) for each individual study. The majority of the values are positive, indicating that each of these trials reported a beneficial effect of extracorporeal shockwave therapy (ESWT) on ulcer healing. To make this easier to understand, the Z-value tells us how far a study’s result is from being due to chance alone; a higher value indicates a stronger result. The *p*-value (in the “Sig.” column) gives us the probability that the results were obtained by chance. We observe that studies such as Omar [27], Snyder [24], and Vangaveti [23] showed a statistically significant effect on their own, with *p*-values below 0.05.

Table 4 shows the overall result of the meta-analysis, combining the data from all studies. The average odds ratio was OR = 2.851 (with a 95% confidence interval of [1.938, 4.193]). In simple terms, the Odds Ratio (OR) compares the likelihood of an event (in this case, ulcer healing) in the treatment group versus the control group. An OR of 2.851 is highly significant; it implies that patients receiving ESWT are 2.851 times more likely to achieve complete ulcer closure compared to the control group. This result is statistically significant (z = 5.320, *p* < 0.001), demonstrating a robust effect.

**Figure 4:**
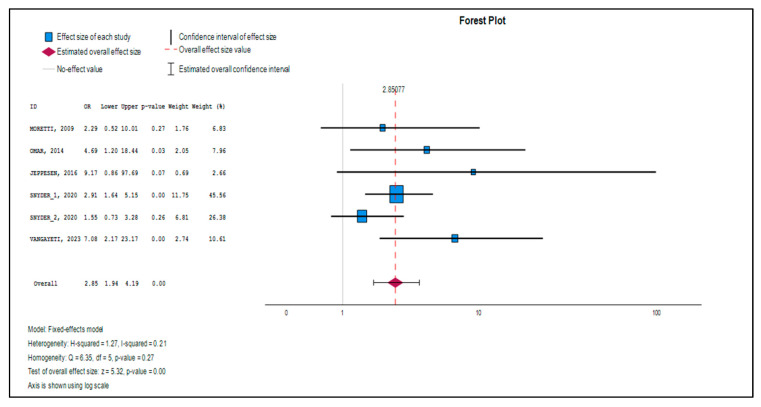
Forest plot [23,24,25,26,27].

Figure 4 (Forest Plot) complements Table 4 by visually representing the results of each study and the combined effect. In this type of graph, each study is represented by a square and its horizontal line (the confidence interval). If a study shows no significant effect, its confidence line will cross the central line (which represents an OR = 1). The “diamond” at the bottom of the plot, which represents the overall effect, is clearly located to the right of the null value (OR = 1), and its ends do not touch this line. This visually confirms the statistical significance of the overall result, indicating that the beneficial effect of ESWT is real and not a random outcome.

### 3.4. Heterogeneity Analysis

Heterogeneity refers to the true differences between studies that could affect the results (e.g., differences in patient type, ulcer severity, or treatment protocol). To evaluate if the results of the individual studies are consistent with each other, we performed heterogeneity tests. The Q-test (Table 5) did not show statistically significant heterogeneity (Q(5) = 6.351, *p* = 0.274). In this case, a *p*-value greater than 0.05 suggests that there is no notable difference between the study effects, which allows us to safely combine them.

**Figure 5:**
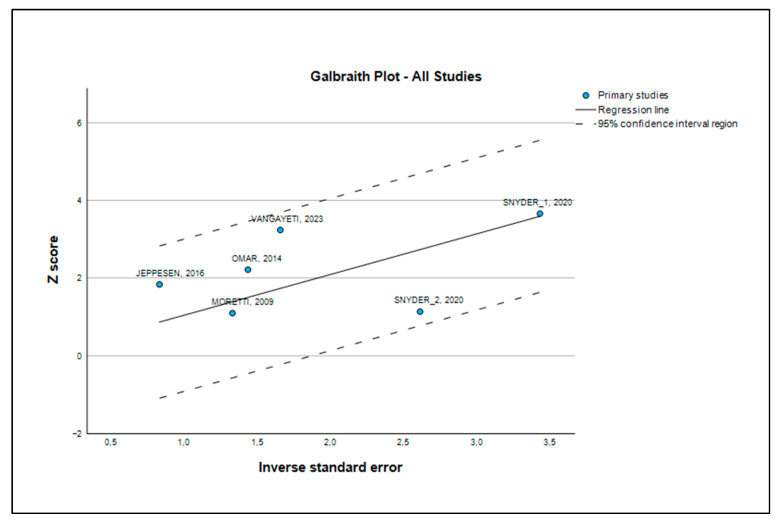
Galbraith plot [23,24,25,26,27].

**Figure 6:**
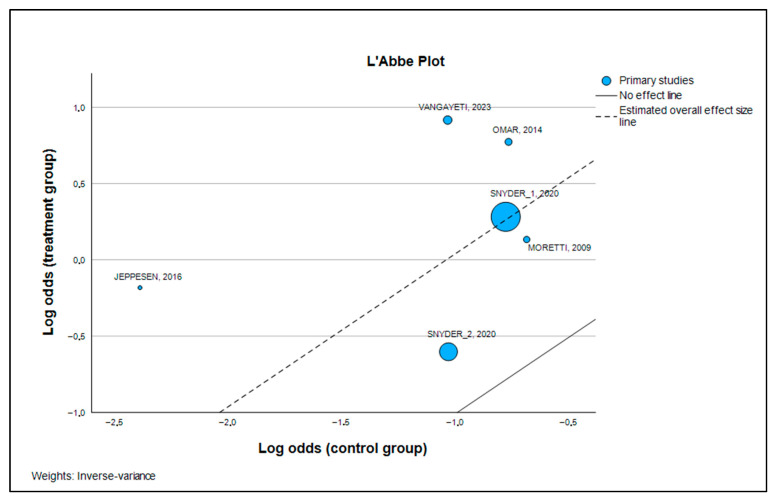
L’Abbé plot [23,24,25,26,27].

Table 6 presents the heterogeneity measures in a more quantitative way. The I2 test yielded a value of 21.3% with a 95% confidence interval of [0.0%, 65.7%]. The I2 value is interpreted as the percentage of the total variability in the study results that is due to true heterogeneity (clinical or methodological differences) rather than chance. A value of 21.3% is low, reinforcing our conclusion that the results are quite homogeneous. The Galbraith plot (Figure 5) provided visual confirmation of the homogeneity, as all studies were located within two standard deviations of the regression line. Similarly, the L’Abbé plot (Figure 6) clearly illustrated that in all trials, ESWT consistently outperformed standard care, as all data points were situated above and to the left of the line of equality.

### 3.5. Publication Bias and Robustness Evaluation

**Figure 7:**
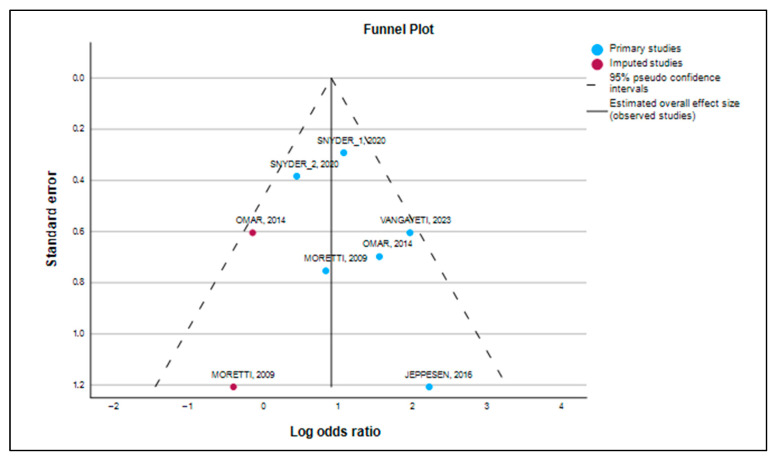
Funnel plot [23,24,25,26,27].

Publication bias is the tendency for studies with positive results to be more likely to be published than those with negative or null results. To address this potential influence, we performed multiple tests. Figure 7 (Funnel Plot) gives us a first visual impression of this bias. Although the plot showed a slight visual asymmetry, with an apparent lack of studies in the lower-left region (indicating that small studies with negative results may not have been published), the formal tests did not confirm it.

The absence of bias was supported by the Kendall’s tau rank correlation test (Table 7), which did not detect asymmetry in the funnel plot (tau = −0.333, *p* = 0.200). This *p*-value, being greater than 0.05, indicates that the correlation between the study size and its effect size is not significant, which is a good indication of the absence of bias.

The formal Egger’s test (Table 8) with an intercept of 0.488 and a *p*-value of *p* = 0.385 (>0.05) confirmed that there is no statistically significant publication bias. If the *p*-value of the Egger’s test had been less than 0.05, it would have indicated that publication bias is likely. In our case, the result is reassuring.

**Figure 8:**
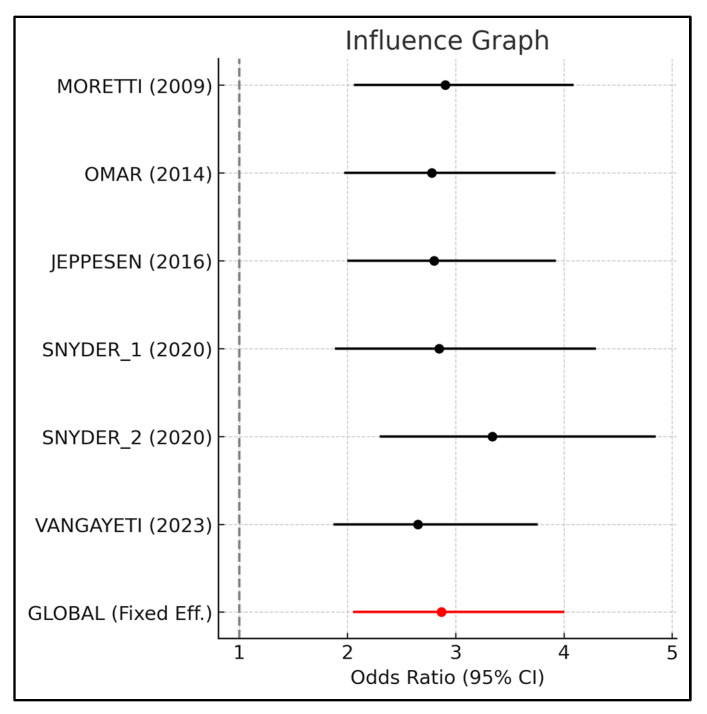
Influence graph [23,24,25,26,27].

The sensitivity analysis (Table 9 and Figure 8) also confirmed that our findings are robust. This analysis evaluates the stability of the overall result by removing each study one by one. Table 9 shows the meta-analysis results when each study is omitted individually. It was observed that the combined odds ratio remained consistently above the null value (OR = 1), which demonstrates that no single study has a disproportionate influence on the result.

### 3.6. Trim-and-Fill Analysis

Finally, the “Trim-and-Fill” analysis (Table 10) suggested the possible existence of two missing studies to achieve symmetry in the funnel plot. However, even after their hypothetical inclusion, the adjusted effect size (Log OR = 0.900, OR = 2.461) remained very close to the original effect size (Log OR = 1.048, OR = 2.851). This confirms that our findings are consistent and that the beneficial effect of ESWT is unlikely to be due to publication bias.

## 4. Discussion

The results of this meta-analysis provide strong and compelling evidence on the efficacy of extracorporeal shockwave therapy (ESWT) as an adjunctive treatment for chronic diabetic foot ulcers (DFU). The main finding is that ESWT significantly increases the complete ulcer healing rate compared to standard of care (SOC) alone, with an overall odds ratio (OR) of 2.851. This result is not only statistically significant (*p* < 0.001) but also clinically very relevant, suggesting that patients who receive ESWT are almost three times more likely to achieve complete ulcer closure than those in the control group. This therapy represents a promising advance in the management of a diabetic complication that is often difficult to treat and has a significant impact on a patient’s quality of life.

### 4.1. ESWT as an Adjunctive Therapy in the Treatment of Diabetic Foot Ulcers

The standard of care (SOC) for diabetic foot ulcers is a multifaceted approach that includes aggressive debridement, infection control, revascularization if necessary, biomechanical load management through offloading, and meticulous wound care. However, despite these efforts, a considerable percentage of DFUs become stagnant, leading to a prolonged healing process and increasing the risk of amputation. It is at this point that adjunctive therapies, such as ESWT, become crucial, as they aim to reactivate the healing process when conventional treatments are not enough.

Our findings align with and expand on previous research on ESWT and wound healing. While other meta-analyses on this topic exist, the inclusion of more recent randomized controlled trials (RCTs), such as those by Snyder [24] and Vangaveti [23], provides a more current and robust body of evidence. The consistency of our results with previous studies underscores the reliability of the therapy. A recent meta-analysis by Huang [28] and the systematic review by Wu [29] also found that ESWT improves healing rates and reduces healing time in DFUs, reinforcing our conclusion. This strengthens the case for ESWT in the management of DFUs that often do not respond to conventional treatments.

### 4.2. Molecular and Cellular Mechanisms: A Deeper Look

The efficacy of ESWT in wound healing goes beyond a simple mechanical effect. Shockwaves induce a complex biological response at the cellular level. They activate mechanotransduction, a process in which cells convert mechanical stimuli into biochemical responses. This leads to the release of a series of mediators that re-engage the wound healing signaling pathways. Research suggests that ESWT promotes angiogenesis (the formation of new blood vessels) by increasing the expression of growth factors like vascular endothelial growth factor (VEGF) and nitric oxide, which improves microcirculation and oxygen supply to the ischemic tissue [29,30]. In addition to angiogenesis, ESWT has been shown to have anti-inflammatory and antibacterial effects. It modulates the immune response, reducing the chronic inflammation that often hinders the healing of DFUs. Shockwaves have also been observed to weaken bacterial membranes, which could reduce the bioburden in the wound, a critical factor in non-healing ulcers [28]. The therapy may also mitigate pain, a common issue for patients, as it is believed to affect sensory nerves and nociceptors in the fascia [31].

### 4.3. ESWT in the Context of Other Adjunctive Therapies

Current clinical practice guidelines, such as those from the International Working Group on the Diabetic Foot (IWGDF), do not yet recommend ESWT as a routine treatment due to uncertainties about dosing and patient selection [28]. However, this is changing as evidence accumulates. ESWT compares favorably with other advanced therapies. For example, studies have shown that it has a higher healing rate than hyperbaric oxygen therapy (HBOT) and negative pressure therapy [32]. A recent meta-analysis by Huang [28] and the systematic review by Wu [29] support these findings, showing that ESWT is an effective alternative when other therapies fail.

### 4.4. Strengths and Robustness of the Results

One of the main strengths of our analysis is the low heterogeneity observed among the included studies. Unlike other meta-analyses that often find large differences between studies, our I2 value of 21.3% and a non-significant Q-analysis (*p* = 0.274) indicate that the variability in the study results is primarily due to chance (sampling variance) rather than significant clinical or methodological differences between the trials (Table 5 and Table 6). This homogeneity is crucial, as it allows for a more reliable combination of data, increasing the validity of our estimate of the combined effect size.

The visual confirmation from the Galbraith plots (Figure 5) and L’Abbé plots (Figure 6) further reinforces this conclusion. Furthermore, our analysis demonstrated the robustness of the results and a low risk of publication bias. Publication bias is a common problem in research, where studies with positive results are more likely to be published than those with negative results. To address this, we used several statistical tests. Egger’s test, with a non-significant *p*-value of 0.385, and Kendall’s tau rank correlation test, with a *p*-value of 0.200, suggest the absence of a statistically significant publication bias (Table 7 and Table 9).

Although the funnel plot (Figure 8) showed a slight visual asymmetry, the “Trim-and-Fill” analysis (Table 10) identified a theoretical number of two missing studies to achieve perfect symmetry. However, even after their hypothetical inclusion, the adjusted odds ratio remained very close to our main finding (OR = 2.461 vs. OR = 2.851). This confirms that our results are not an artifact of selective publication but a genuine reflection of the efficacy of ESWT. The stability of our findings was also demonstrated in the sensitivity analysis (Table 8), which showed that the overall effect size remained consistent even when individual studies were omitted, underscoring the reliability of our results.

### 4.5. Cost-Effectiveness and Clinical Considerations

A crucial aspect for the widespread adoption of any new therapy is its cost-effectiveness. While the literature on ESWT for DFU is limited in this area, feasibility studies suggest it can be an economically viable option [32]. While it has an upfront cost, it can reduce long-term costs by accelerating healing, decreasing the need for hospitalizations, reducing the frequency of dressing changes, and, most importantly, preventing costly and life-altering amputations. In terms of patient selection, research indicates that the therapy may be particularly beneficial for chronic ulcers that do not respond to standard treatment. Patients with type 1 diabetes and non-smokers seem to have a better response to treatment, suggesting that specific patient and lifestyle factors may influence the outcome [31].

### 4.6. Limitations and Future Directions

Despite these strengths, it is important to acknowledge the limitations of this meta-analysis. While the included studies are methodologically sound, they varied in key aspects such as patient characteristics (e.g., DFU classification) and ESWT application protocols. The lack of standardization in treatment protocols (such as dose, frequency, and duration of therapy) remains a limitation in the literature, as noted by other researchers [33]. Although our heterogeneity tests did not detect significant differences, these factors could subtly influence the results. In the future, RCTs should strive to adopt standardized treatment protocols to allow for more direct comparisons and increase the certainty of the evidence.

Despite the growing evidence of its efficacy, ESWT has not yet been routinely incorporated into the clinical practice guidelines of many organizations, such as the International Working Group on the Diabetic Foot (IWGDF) [33]. Uncertainty about the optimal dose, patient selection, and long-term impact are barriers to its widespread adoption. Therefore, future research should focus not only on efficacy but also on optimizing ESWT protocols for different types of ulcers, as well as exploring its long-term outcomes and cost-effectiveness in various healthcare settings.

## 5. Conclusions

This meta-analysis provides compelling evidence that ESWT is a very effective complementary therapy for the treatment of chronic DFUs. Our findings demonstrate a robust and statistically significant benefit, supported by a low risk of bias and high consistency of results among the studies. This suggests that ESWT should be considered a valuable addition to the standard of care for patients with chronic DFUs, and that future research must address the existing gaps in protocol standardization to facilitate its clinical implementation.

## Figures and Tables

**Table 1:** Summary of individual trials, by intervention strategy.

First Author, Year of Publication, Country	N, Gender (M/F)	Age (Mean ± SD)	HbA1c (Mean ± SD)	BMI (Mean ± SD)	Average Size	Years Diabetes	Ulcer Classification	Treatment Strategy	ESWT Application	**Time Treatment**	**Healing** **Rate**	**Conclusions**
**Vangaveti et al.,** **2023,** **Australian** **[23]**	N = 48 (34/14) E = 25 (17/8) C = 23 (17/6)	N = 62.0 (52.3–78.5) E = 62 (52–78) C = 62 (52–79)	C = 7.8 E = 8.1	N = 35.5 (29.7–39.5) E = 36.4 ± 6.5 C = 33.4 ± 7.4	C = 48 (20–408) E = 70 (25–226)	-	Texas Classification: A1 or higher	Effects of ESWT on wound healing in patients with DFU undergoing SOC	Sterile ultrasound gel was applied to the wound, and a sterile foil was placed on top to ensure no air bubbles were present in the treatment area. This was followed by the application of ultrasound gel followed by ESWT. The dosage regimen was administered as 0.11 mJ/mm^2^ at 5 Hz and an interval of 100 SW with pressure head between 6 and 10. The number of pulses was increased based on wound’s size with a minimum of 310 pulse/cm^2.^	6 w	E = 5 (20%) C = 6 (26.1%)	ESWT healing rate did not reach statistical significance at 6 weeks
**Snyder et al.,** **2020,** **US** **[24]**	N = 206 (137/69) E = 107 (83/24) C = 99 (54/45)	E = 60.4 ± 10.4 C = 56.2 ± 9.4	-	E = 31.8 ± 5.1 C = 31.6 ± 5.2	E = 35 ± 32 C = 28 ± 24	E = 18.0 ± 10.0 C = 15.7 ± 11.1	Texas Classification:1A or 2A	Efficacy of ESWT compared with SOC.	First application of 500 impulses lasting between 2 and 5 min using an energy flux density of 0.23 mJ/mm^2^ at a rate of 4 impulses/seg and delivered at a power setting of E^2^. Hereafter, subjects received SOC and 3 additional adjunctive active or sham device applications every 3 ± 1 days over 2 w.	12 w	E = 61 (47.7%) C = 31 (31.3%)	ESWT is an effective for neuropathic DFU that do not respond to standard care alone.
**Snyder et al.,** **2020,** **US** **[24]**	N = 130 (103/27) E = 65 (54/11) C = 65 (49/16)	E = 59.1 ± 9.4 C = 56.8 ± 10.7	-	E = 31.4 ± 5.6 C = 31.6 ± 5.5	E = 3.71 ± 2.8 C = 3.73 ± 2.8	E = 15.44 ± 11.4 C = 17.45 ± 13.3	Texas Classification:1A or 2A	Efficacy of ESWT compared with SOC.	ESWT was from a dermaPACE device. Outpatient care procedure with no anesthesia. Ulcer was covered with a sterile cellulose barrier. The treatment dosage was ulcer size dependent with the numbers of impulses equal to the treatment area in cm2 × 8, with a minimum of 500 impulses at energy setting E2 at a rate of 4 shocks/seg. Area was calculated by extending the actual perimeter of the ulcer for 1.0 cm in all directions. The treatments were conducted two times/w for 3 weeks for a total of 6 treatments.	10 w	E = 23 (35.4%) C = 17 (26.2%)	ESWT is an effective for neuropathic DFU that do not respond to standard care alone.
**Moretti et al.,** **2009,** **Italy** **[25]**	N = 30 (16/14) E = 15 (9/6) C = 15 (7/8)	E = 56.2 ± 4.9 C = 56.8 ± 7.5	-	-	E = 297.8 ± 129.4 C = 245 ± 100.9 mm^2^	-	Wagner 2, 3 and 4	Evaluate if ESWT is effective in the management. of neuropathic diabetic foot ulcers to compare SOC.	Three sessions (every 72 h), with 100 pulses per 1 cm^2^ of wound delivered at each session at a flux density (0.03 mJ/mm^2^) using an electromagnetic lithotripter with a cylindrical coil, parabolic focus, and ultrasound scanning.	20 w	E = 8 (53.3%) C = 5 (33.3%)	ESWT may be a useful adjunct in the management of diabetic foot. ulceration.
**Jeppesen et al.,** **2016,** **Denmark** **[26]**	N = 23 (16/7) E = 11 (5/6) C = 12 (11/1)	E = 65.3 ± 12.9 C = 67.8 ± 9.7	E = 8.6 ± 0.8 C = 7.7 ± 1.2	E = 27.0 ± 5.4 C = 26.3 ± 3.7	-	E = 16.3 ± 12.2 C = 25.1 ± 15.0	Wagner 1 and 2	Efficacy of ESWT on healing DFU compared to SOC.	6 ESWT treatments over 3 w. Treatments were carried out with a DUOLITH SD1 T-top shockwave device delivering focused shockwaves with energy flux density 0.2 mJ/mm^2^ and frequency 5 Hz. Ulcer surface and perimeter of ulcer extending 1 cm in every direction was treated with ESWT, using 250 shocks/cm^2^ and focal area 0–30 mm. Furthermore, 500 deep shocks focal area (15–45 mm) were applied on the anatomical location of arteries supplying ulcer location.	3 w	E = 5 (34.5%) C = 1 (5.6%)	Potential beneficial effect of ESWT on ulcer healing as well as tissue oxygenation.
**Omar et al.,** **2014,** **Egypt** **[27]**	N = 38 (27/11) E = 19 (14/5) C = 19 (13/6)	E = 56.59 ± 7.35 C = 57.0 ± 5.39	E = 8.92 ± 1.93 C = 8.22 ± 1.90	E = 27.44 ± 2.57 C = 27.35 ± 3.94	E = 7.89 ± 2.97 C = 8.62 ± 3.47	E = 12.0 ± 3.66 C = 13.14 ± 3.78	Texas Classification:1A or 2A	Evaluate the efficacy of ESWT on the healing rate.	Cleaned saline, removed necrotic tissues, and covered. ESWT at a frequency of 100 pulse/cm^2^, and energy flux density of 0.11 mJ/cm^2^. Twice a week, with one-week interval, and for a total of eight sessions.	8 w	E = 13 (54%) C = 6 (28.5%)	ESWT significant reduction in wound size and median time ulcer healing, with no adverse reactions.

N = Number of participants; M = male; F = female; E = experimental group; C = control group; SD = standard deviation; EWST = Effectiveness of extra corporeal shockwave therapy; SOC = standard of care; w = weeks; DFU = diabetic foot ulcer.

**Table 2:** Points table by CASPe tool for critical reading of scientific evidence.

First Author	P1	P2	P3	P4	P5	P6	P7	P8	P9	P10	P11	TOTAL
Vangaveti et al. [23]	YES	YES	YES	NO	YES	YES	YES	YES	YES	YES	YES	10/11
Snyder et al. [24]	YES	YES	YES	NO	YES	YES	YES	YES	YES	YES	YES	10/11
Snyder et al. [24]	YES	YES	YES	YES	YES	YES	YES	YES	YES	YES	YES	10/11
Moretti et al. [25]	YES	YES	YES	NO	YES	YES	YES	YES	YES	YES	YES	10/11
Jeppesen et al. [26]	YES	YES	YES	YES	YES	YES	YES	YES	YES	YES	YES	10/11
Omar et al. [27]	YES	YES	YES	YES	YES	YES	YES	YES	YES	YES	YES	10/11

**Table 3:** Effect Size Estimates for Individual Studies.

ID	Effect Size	Std. Error	Z	Sig. (2-Tailed)	95% Confidence Interval	
					Lower	Upper
Moretti et al. [25]	0.827	0.7536	1.097	0.273	−0.650	2.304
Omar et al. [27]	1.546	0.6980	2.215	0.027	0.178	2.914
Jeppesen et al. [26]	2.216	1.2073	1.835	0.066	−0.151	4.582
Snyder et al. [24]	1.068	0.2917	3.660	<0.001	0.496	1.640
Snyder et al. [24]	0.436	0.3833	1.137	0.256	−0.316	1.187
Vangaveti et al. [23]	1.958	0.6046	3.238	0.001	0.773	3.143

**Table 4:** Effect Size Estimates.

	Effect Size	Std. Error	Z	Sig. (2-Tailed)	95% Confidence Interval	
					Lower	Upper
Overall	0.048	0.1969	5.320	<0.001	0.662	1.434

**Table 5:** Q-test for heterogeneity.

	Chi-Square (Q Statistic)	df	Sig.
Overall	6.351	5	0.274

**Table 6:** Heterogeneity Measures.

		Index	95% Confidence Interval	
			Lower	Upper
Overall	H-squared	1.270	0.553	2.916
	I-squared (%)	21.3	0.0	65.7

**Table 7:** Rank correlation test for funnel plot asymmetry.

Kendall’s Tau	*p*
−0.333	0.200

**Table 8:** Egger’s regression-based test.

Egger’s Regression-Based Test ^a^						
**Parameter.**	Coefficient	Std. Error	t	Sig. (2-Tailed)	95% Confidence Interval	
					Lower	Upper
(Intercept)	0.488	0.5005	0.974	0.385	−0.902	1.877
SE ^b^	1.281	1.0377	1.234	0.285	−1.600	4.162

a. Fixed effects multiplicative meta-regression; b. Standard error of effect size.

**Table 9:** Sensitivity analysis—Fixed effects model.

				CI (95.0%)		
Omitted Study	Year	*n*	OR	Lower Limit	Upper Limit	Relative Change (%)
Moretti et al. [25]	2009	642	2.9031	2.0601	4.0911	1.23
Omar et al. [27]	2014	634	2.7795	1.9694	3.923	−3.08
Jeppesen et al. [26]	2016	649	2.8008	1.9985	3.9252	−2.34
Snyder et al. [24]	2020	466	2.8467	1.8859	4.2971	−0.73
Snyder et al. [24]	2020	542	3.3396	2.2997	4.8496	16.45
Vangaveti et al. [23]	2023	614	2.6523	1.8723	3.7573	−7.51
GLOBAL		672	2.8678	2.0532	4.0055	

**Table 10:** Effect size estimates for Trim-and-Fill analysis.

	Number	Effect Size	Std. Error	Z	Sig. (2-Tailed)	95% Confidence Interval			95% Confidence Interval	
						Lower	Upper	Exp. Effect Size	Lower	Upper
Observed	6	1.048	0.1969	5.320	<0.001	0.662	1.434	2.851	1.938	4.193
Observed + Imputed ^a^	8	0.900	0.1850	4.867	<0.001	0.538	1.263	2.461	1.712	3.536

a. Number of imputed studies: 2.

## Data Availability

The original contributions presented in this study are included in the article. Further inquiries can be directed to the corresponding author.
